# EMERGENCY PREPAREDNESS AMONG CLIENTS OF OLDER AMERICANS ACT PROGRAMS

**DOI:** 10.1093/geroni/igad104.2895

**Published:** 2023-12-21

**Authors:** Joanne Campione, Kristen Robinson

**Affiliations:** Westat, Durham, North Carolina, United States; Administration for Community Living, Washington, District of Columbia, United States

## Abstract

Older adults who are clients of Older Americans Act (OAA) programs, such as home-delivered meals and transportation services, often have unique needs during an emergency or crisis. Many OAA clients have mobility challenges and/or chronic health conditions, making it difficult to access and respond to emergency directions. For these reasons, in 2022, the Administration for Community Living added to the annual National Survey of Older Americans Act Participants (NSOAAP) a one-time module of questions regarding emergency preparedness. Among 5,136 respondents of the 2022 NSOAAP, 67% said they have ready 3 days’ worth of food and water, 69% have a way to charge their phone without electricity, and 51% have a battery-powered radio. Thirty-seven percent of the clients said their household has a medical device that requires electricity. While 74% said they have a list of list of family, friends, and community organizations, only 37% said their household has a specific plan for a disaster or emergency. Seven percent said they would not evacuate if public authorities announced a mandatory evacuation with the top three reasons for not evacuating being: 1) concern about leaving pets, 2) lack of transportation, and 3) could not be moved due to health problems. In summary, support services that an older adult relies on to live at home, such as help from family caregivers and in-home health care, may be unavailable due to a disaster thus increasing a person’s vulnerability and possibly leading to nursing home care that may have been otherwise avoidable.

